# The Pseudomonas stutzeri-Specific Regulatory Noncoding RNA NfiS Targets *katB* mRNA Encoding a Catalase Essential for Optimal Oxidative Resistance and Nitrogenase Activity

**DOI:** 10.1128/JB.00334-19

**Published:** 2019-09-06

**Authors:** Hongyang Zhang, Yuhua Zhan, Yongliang Yan, Yichao Liu, Guihua Hu, Shanshan Wang, Hua Yang, Xuemeng Qiu, Yaqun Liu, Jiang Li, Wei Lu, Claudine Elmerich, Min Lin

**Affiliations:** a Biotechnology Research Institute/National Key Facility for Crop Gene Resources and Genetic Improvement, Chinese Academy of Agricultural Sciences, Beijing, China; b Institut Pasteur, Paris, France; University of Illinois at Urbana-Champaign

**Keywords:** H_2_O_2_, NfiS, *Pseudomonas stutzeri* A1501, *katB* mRNA, nitrogen fixation, oxidative stress response, regulatory ncRNA

## Abstract

Pseudomonas stutzeri A1501 is a versatile nitrogen-fixing bacterium capable of living in diverse environments and coping with various oxidative stresses. NfiS, a regulatory noncoding RNA (ncRNA) involved in the control of nitrogen fixation in A1501, was previously shown to be required for optimal resistance to H_2_O_2_; however, the precise role of NfiS and the target genes involved in the oxidative stress response is entirely unknown. In this work, we systematically investigated the NfiS-based mechanisms underlying the response of this bacterium to H_2_O_2_ at the cellular and molecular levels. A mutant strain carrying a deletion of *nfiS* showed significant downregulation of oxidative stress response genes, especially *katB*, a catalase gene, and *oxyR*, an essential regulator for transcription of catalase genes. Secondary structure prediction revealed two binding sites in NfiS for *katB* mRNA. Complementation experiments using truncated *nfiS* genes showed that each of two sites is functional, but not sufficient, for NfiS-mediated regulation of oxidative stress resistance and nitrogenase activities. Microscale thermophoresis assays further indicated direct base pairing between *katB* mRNA and NfiS at both sites 1 and 2, thus enhancing the half-life of the transcript. We also demonstrated that *katB* expression is dependent on OxyR and that both OxyR and KatB are essential for optimal oxidative stress resistance and nitrogenase activities. H_2_O_2_ at low concentrations was detoxified by KatB, leaving O_2_ as a by-product to support nitrogen fixation under O_2_-insufficient conditions. Moreover, our data suggest that the direct interaction between NfiS and *katB* mRNA is a conserved and widespread mechanism among P. stutzeri strains.

**IMPORTANCE** Protection against oxygen damage is crucial for survival of nitrogen-fixing bacteria due to the extreme oxygen sensitivity of nitrogenase. This work exemplifies how the small ncRNA NfiS coordinates oxidative stress response and nitrogen fixation via base pairing with *katB* mRNA and *nifK* mRNA. Hence, NfiS acts as a molecular link to coordinate the expression of genes involved in oxidative stress response and nitrogen fixation. Our study provides the first insight into the biological functions of NfiS in oxidative stress regulation and adds a new regulation level to the mechanisms that contribute to the oxygen protection of the MoFe nitrogenase.

## Abstract (precis)

Protection against oxygen damage is crucial for survival of nitrogen-fixing bacteria due to the extreme oxygen sensitivity of nitrogenase. This work exemplifies how the small ncRNA NfiS coordinates oxidative stress response and nitrogen fixation via base pairing with *katB* mRNA and *nifK* mRNA. Hence, NfiS acts as a molecular link to coordinate the expression of genes involved in oxidative stress response and nitrogen fixation. Our study provides the first insight into the biological functions of NfiS in oxidative stress regulation and adds a new regulation level to the mechanisms that contribute to the oxygen protection of the MoFe nitrogenase.

## INTRODUCTION

Members of the genus *Pseudomonas* show a versatile metabolic capacity (such as denitrification, degradation of aromatic compounds, and nitrogen fixation) and broad potential for adaptation (1). *Pseudomonas* strains are found in diverse environments, where they encounter various endogenous and exogenous oxidative stresses. Specific physiological responses to reactive oxygen species have been well characterized in some *Pseudomonas* strains (2). Most *Pseudomonas* strains are obligate aerobes that produce metabolic energy through aerobic respiration. H_2_O_2_ can be provided externally by redox-cycling agents or host factors or by incomplete reduction of O_2_ during aerobic respiration (3). During aerobic respiration, incomplete reduction of O_2_ can lead to the production of H_2_O_2_ (3). Because it freely diffuses into cells, H_2_O_2_ oxidizes proteins, nucleic acids, and lipids and thus can damage the cell (4). The rapid response of most *Pseudomonas* strains to H_2_O_2_ is governed by the global activator OxyR (5). OxyR senses the redox status and activates the transcription of the multiple catalase genes (6). Catalase is a key component of antioxidant defenses, forming the first line of defense against excess H_2_O_2_.

Pseudomonads were shown to cope with oxidative stresses in almost all environments by modulating the gene expression of sophisticated defense systems. The LysR-type regulator OxyR, a central peroxide sensor of the oxidative stress response in bacteria, has been extensively studied in Pseudomonas aeruginosa (5, 7, 8). OxyR regulates the transcription of defense genes in response to a low level of cellular H_2_O_2_ (7). In the presence of H_2_O_2_, OxyR undergoes rearrangement of its secondary structures by forming an intramolecular disulfide bond, resulting in oxidized OxyR (9). Oxidized OxyR stimulates the expression of two major catalase structural genes, *katA* and *katB*, which encode KatA and KatB, and the peroxiredoxin gene *ahpC*, which encodes AhpC (5). Several additional global regulators are also implicated in the control of catalase expression, including the Las and Rhl quorum-sensing systems (10), RpoS (11), and the iron uptake regulator Fur (12). These overlapping regulatory networks allow *Pseudomonas* to coordinate oxidative stress defense systems in response to different environmental stresses.

Regulatory noncoding RNAs (ncRNAs), also referred to as small RNAs (sRNAs), are implicated in oxidative stress response systems and have been extensively studied in Escherichia coli and pseudomonads (13, 14). One of the first characterized sRNAs in E. coli was the oxidative stress-induced OxyS (15). This sRNA works in concert with OxyR to coordinate the expression of catalase genes at the posttranscriptional level and links the oxidative stress response to more global responses (16). In addition, three sRNAs (termed DsrA, RprA, and ArcZ) positively regulate the translation of *rpoS* under low-temperature stress and osmotic shock and in response to aerobic/anaerobic growth conditions, respectively (17–19). P. aeruginosa PrrF1 and PrrF2 have overlapping roles in the negative regulation of genes involved in diverse functions, including iron storage, defense against oxidative stress, and intermediary metabolism (20). In both P. aeruginosa and P. fluorescens, several GacA-controlled Rsm ncRNAs regulate the response to oxidative stress and the expression of extracellular products, including biocontrol factors, while RgsA ncRNA, which is activated by GacA, contributes to resistance to H_2_O_2_ (21).

Biological nitrogen fixation, defined as the reduction of N_2_ to ammonia by nitrogenase, is an energy-expensive process. Oxygen supports sufficient production of ATP for the nitrogen fixation process but can also rapidly limit the activity of the nitrogenase and repress its synthesis. Thus, nitrogen-fixing bacteria have to evolve various strategies to meet the energy demands of nitrogen fixation while protecting nitrogenase from oxygen damage. Members of the genus *Pseudomonas* exhibit remarkable metabolic and physiologic versatility, enabling colonization of a wide range of abiotic and biotic environments. Surprisingly, nitrogen fixation is a rare feature in the genus *Pseudomonas*, and so far, most isolated strains are phylogenetically affiliated with Pseudomonas stutzeri (22–25). Determination of the nitrogen-fixing P. stutzeri genome sequence led to the identification of a highly conserved nitrogen-fixing island acquired by horizontal gene transfer (24, 26). P. stutzeri A1501, isolated from the rice rhizosphere in southern China, exists either in a free-living lifestyle in the soil or in root association with host plants. This bacterium has emerged as a model organism to study the global regulation of nitrogen fixation and molecular interactions between nitrogen-fixing bacteria and host plants and was shown to improve plant growth and plant nitrogen content using a ^15^N dilution technique under two water regimen conditions (27). Similar to most diazotrophs, A1501 can fix nitrogen only under microaerobic conditions and exhibits optimal nitrogenase activity at an oxygen concentration of 1.0% (22). The genes responsible for the oxygen protection mechanisms in A1501 have yet to be determined. Previous studies have demonstrated that a novel P. stutzeri-specific ncRNA, NfiS, whose synthesis was dramatically increased under nitrogen fixation conditions, plays a crucial role in optimizing nitrogen fixation via base pairing with the nitrogenase gene *nifK* mRNA (28). An *nfiS* mutant strain was more sensitive to 20 mM H_2_O_2_ than the wild-type (WT) strain, whereas the overexpression of NfiS led to enhanced resistance, suggesting that NfiS is essential for optimal resistance to oxidative stress. However, its target genes under oxidative stress have yet to be defined, and the regulatory mechanisms involved in the oxidative stress response remain unknown. In this study, we show that *nfiS* mutation led to significant downregulation of oxidative stress response genes, especially *katB* and *oxyR*. Furthermore, rapid adaptive stress responses and efficient protection of nitrogenase against oxidative injuries in A1501 were experimentally confirmed by mutant strain construction and complementation experiments. We report here that NfiS regulates optimal oxidative stress resistance via direct binding with *katB* mRNA, which results in enhanced stability of the transcript, or via the indirect transcriptional activation of OxyR. This global regulation might be a conserved and widespread mechanism in P. stutzeri strains.

## RESULTS

### NfiS deletion caused the decrease in expression of the oxidative stress response genes.

**FIG 1:**
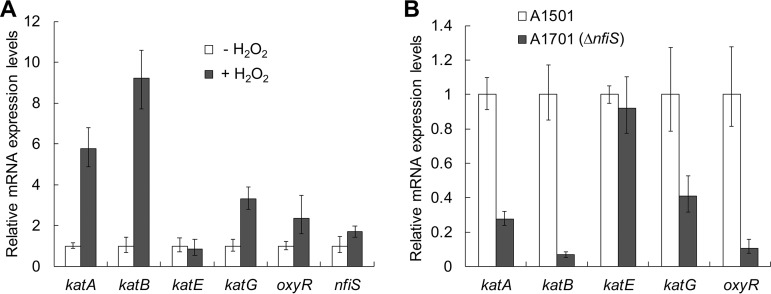
qRT-PCR analysis of the relative expression levels of five selected oxidative stress-responsive genes and *nfiS*. (A) Gene expression of A1501 grown in LB for 10 min with or without H_2_O_2_ (12 mM). (B) Gene expression of A1501 and *nfiS* mutant A1701 grown in LB with H_2_O_2_. Error bars show the standard deviation from three independent experiments.

We previously reported that an *nfiS* deletion mutant strain (A1701) displayed increased sensitivity to H_2_O_2_-induced oxidative stress (28), implying that this ncRNA might act as a regulator of the oxidative stress response. The A1501 genome contains four catalase genes (*katA*, *katB*, *katE*, and *katG*) and a peroxide sensor gene, *oxyR*, which are highly conserved among different *Pseudomonas* strains **(**see Table S1 in the supplemental material), suggesting common regulatory mechanisms (5, 29). The effect of H_2_O_2_ shock on gene expression level was then determined in LB medium. In the wild-type cells, all selected genes, except *katE*, showed a significant increase after H_2_O_2_ shock (Fig. 1A). This is in agreement with the observation that increasing the concentration of H_2_O_2_ from 0 mM to 12 mM was correlated with an increase in the total catalase activity, with optimal activity observed at a concentration of 4.0 mM H_2_O_2_ (see Fig. S1 in the supplemental material). In particular, the most dramatic increase (>9.0-fold) was noted for *katB* (Fig. 1A), suggesting a functional relevance to oxidative stress. In the A1701 Δ*nfiS* mutant strain, except for *katE*, all oxidative stress response genes were significantly downregulated, especially *katB* and *oxyR*, whose expression was reduced by 15 and 10 times, respectively (Fig. 1B), suggesting that expression of these genes is NfiS dependent via unknown mechanisms. Computational target prediction using the online tool sTarPicker revealed two possible NfiS binding sites to *katB* mRNA, which were located on the typical stem-loop structures (see Fig. S2A and B in the supplemental material). The predicted site 1 extends from positions 6 to 23 of NfiS and is complementary to the 5′ translated region between nucleotides (nt) 62 and 79 of *katB* mRNA, while the predicted site 2 (from 141 to 153) is complementary to nucleotides 66 to 78 of *katB* mRNA (Fig. S2C and D). In contrast, no possible NfiS binding sites were identified in other selected genes’ mRNAs. We thus hypothesized that NfiS regulated optimal oxidative stress resistance via direct binding with *katB* mRNA.

### Two base-pairing regions contribute to NfiS regulation of oxidative stress resistance.

**FIG 2:**
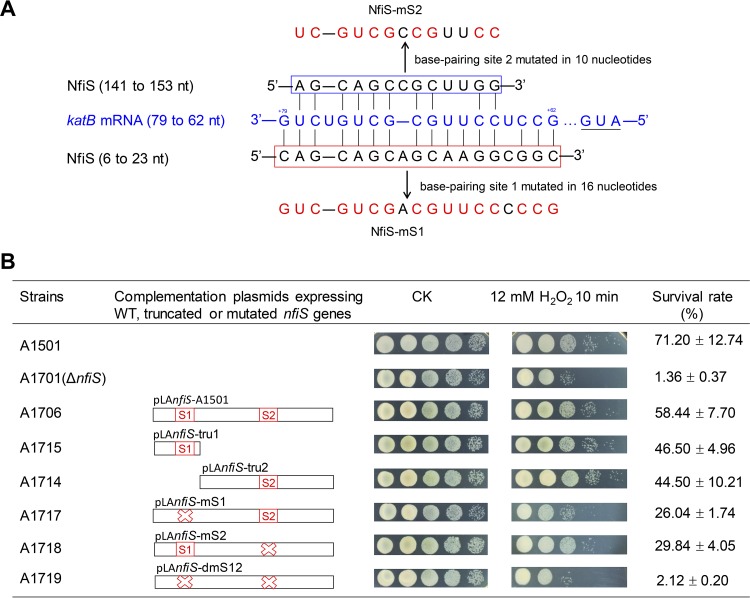
Construction and functional analysis of complementation plasmids expressing WT and truncated and mutated *nfiS* genes. (A) Region (79 to 62 nt) of the *katB* mRNA showing complementarity to predicted base-pairing site 1 (6 to 23 nt) and site 2 (141 to 153 nt) of NfiS. The site 1 and site 2 sequences are shown in red or blue boxes, respectively, and mutated nucleotides of both sites are highlighted in red. (B) Schematic diagrams of the truncated and mutated *nfiS* constructs and the resulting survival rate. CK, untreated culture control. In the linear diagrams, the white bars represent the truncated and mutated *nfiS* constructs, with the red boxes and crosses indicating the location and mutation of the two predicted sites. The survival rate is expressed as the percentage of the number of colonies in the H_2_O_2_-treated samples compared with that in the untreated A1501 sample used as a control.

As mentioned above, predictions of the interaction between NfiS and *katB* mRNA revealed two base-pairing regions, termed sites 1 and 2 (Fig. 2A). To determine whether the two complementary regions are essential for NfiS regulatory functions, we constructed three complementation plasmids, namely, pLA*nfiS*-A1501 expressing the WT *nfiS* gene, pLA*nfiS*-tru1 expressing a truncated *nfiS* gene carrying the first 54 nucleotides with base-pairing site 1, and pLA*nfiS*-tru2 expressing a truncated *nfiS* carrying the 200 remaining nucleotides (between nucleotides 55 and 254) with base-pairing site 2 (Fig. 2B). We found that expression of the WT or truncated *nfiS* genes partially restored H_2_O_2_ resistance of the *nfiS* mutant to WT levels (Fig. 2B), indicating that the truncated *nfiS* genes with either site 1 or 2 functionally substitute for the WT *nfiS* gene.

Site-directed mutagenesis was performed on the two putative base-pairing sites of NfiS to determine if base-pairing sequences are required for NfiS-mediated regulation. As shown in Fig. 2A, two putative base-pairing sequences, 5′-CAGCAGCAGCAAGGCGGC-3′ and 5′-AGCAGCCGCUUGG-3′ (unpaired nucleotides are underlined), were converted to GUCGUCGACGUUCCCCCG and UCGUCGCCGUUCC, leading to complete mismatches. The resulting complementation plasmids (pLA*nfiS*-mS1, expressing a mutated *nfiS* gene lacking site 1, pLA*nfiS*-mS2, expressing a mutated *nfiS* gene lacking site 2, and pLA*nfiS*-dmS12, expressing a mutated *nfiS* gene with double mutation of two sites) were used for further complementation of Δ*nfiS* (A1701). The results showed that either pLA*nfiS*-mS1 (A1717) or pLA*nfiS*-mS2 (A1718) partially restored oxidative stress resistance to the WT levels, whereas pLA*nfiS*-dmS12 (A1719) with a double mutation of the two sites failed to restore oxidative stress resistance, indicating that each of two sites is functional, but not sufficient, for NfiS-mediated regulation of oxidative stress resistance.

### NfiS interacts directly with *katB* mRNA at two base-pairing sites.

**FIG 3:**
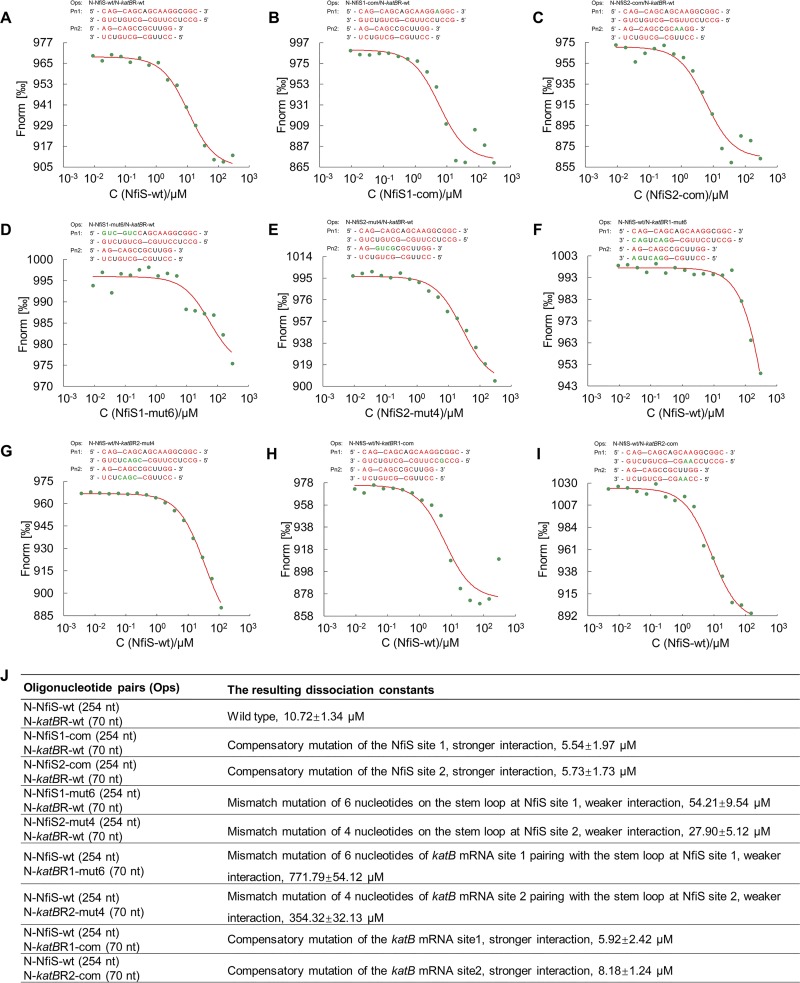
Binding of NfiS to *katB* mRNA. (A to I) The binding affinity of NfiS to *katB* mRNA by MST analysis. Pairing nucleotides are shown in red. Point mutations introduced into synthesized oligonucleotide derivatives are shown in green. (J) Oligonucleotide pairs used for MST analysis and the resulting dissociation constants. See the text for more detail. N, ssRNA oligonucleotides; wt, wild type; mut, mismatch mutation; com, compensatory mutation; NfiS1, NfiS with base-pairing site 1; NfiS2, NfiS with base-pairing site 2; *katB*R1, *katB* mRNA with base-pairing site 1; *katB*R2, *katB* mRNA with base-pairing site 2; Ops, oligonucleotide pairs; Pn1, pairing nucleotides of site 1; Pn2, pairing nucleotides of site 2.

To obtain experimental evidence for the predicted interactions between NfiS and *katB* mRNA, microscale thermophoresis (MST) was used to determine the dissociation constant (*K_d_*) *in vitro* using a set of 254-nt full-length NfiS and 70-nt *katB* mRNA oligonucleotides containing WT or point-mutated base-pairing regions. N-NfiS-wt containing either WT base-pairing site 1 or 2 of NfiS bound to N-*katB*R-wt exhibited dissociation constants (*K_d_*) of 10.72 ± 1.34 μM (Fig. 3A), suggesting a direct interaction of either site 1 or site 2 with *katB* mRNA. We also measured the binding affinity using N-NfiS1-com or N-NfiS2-com oligonucleotides containing compensatory mutations resulting in high match levels to N-*katB*R-wt (Fig. 3B and C). As expected, both compensatory oligonucleotides bound to *katB* mRNA tightly, suggesting stronger interactions. In contrast, N-NfiS1-mut6 and N-NfiS2-mut4 containing the point-mutated base-pairing sequences on stem-loops (33% and 31% mismatch, respectively) of NfiS possessed weaker affinity for *katB* mRNA (Fig. 3D and E). The effect of *katB* mRNA mutations on base pairing with NfiS was investigated using 70 nt of the 5′ translated region of *katB* mRNA containing two mismatch mutations (N-*katB*R1-mut6 and N-*katB*R2-mut4) and two compensatory mutations (N-*katB*R1-com and N-*katB*R2-com). The data indicated that the two mismatch oligonucleotides showed weaker binding affinity for NfiS (Fig. 3F and G), whereas the two compensatory oligonucleotides displayed a stronger interaction with NfiS than the corresponding N-*katB*R-wt oligonucleotides (Fig. 3H and I). The oligonucleotide pairs used for the MST analysis and the resulting dissociation constants observed are reported in Fig. 3J. These data strongly suggest that the mutations in *katB* mRNA have a stronger influence on the *K_d_* for the interaction between NfiS and *katB* mRNA because mutations in the *katB* target site disrupt interactions with both NfiS binding sites, whereas mutations in individual NfiS binding sites still leave the other binding site available for the interaction. Moreover, we observed that the half-life of *katB* mRNA in the *nfiS* mutant was approximately 2-fold less than in A1501, while the half-life of the *nfiS* mutant was restored to the WT level by the complementation plasmid (see Fig. S3 in the supplemental material). These data are in agreement with the base pairing of the two sites of NfiS (sites 1 and 2 defined above [Fig. S2]) with the 5′ translated region of *katB* mRNA, allowing for increased stability of *katB* mRNA.

### NfiS-mediated posttranscriptional regulation of *katB* may be a widespread mechanism among P. stutzeri strains.

**FIG 4:**
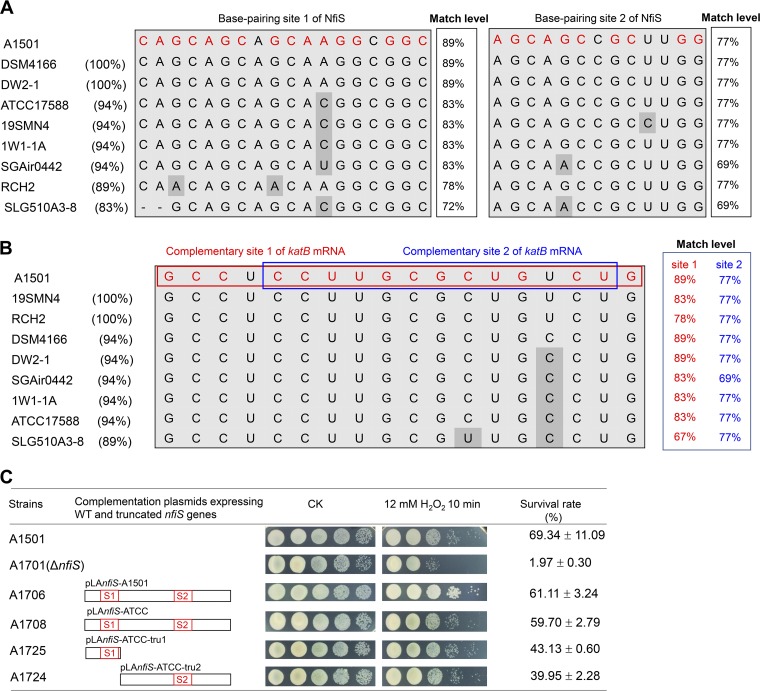
Structural and functional conservation of the two base-pairing sites of NfiS for *katB* mRNA. (A) Sequence alignment of the base-pairing sites in P. stutzeri A1501 NfiS with homologous sequences in different P. stutzeri strains. The nucleotides in the NfiS base-pairing sites matching those of *katB* mRNAs are highlighted in red. Sequence identity of the base-pairing site 1 or 2 identified in the A1501 NfiS and other P. stutzeri strains is shown in parentheses. Match level of base-pairing sequences between NfiS and *katB* mRNA is indicated. (B) Sequence alignment of *katB* mRNA containing the region complementary to P. stutzeri A1501 NfiS in different P. stutzeri strains. Predicted NfiS complementary sites 1 and 2 in *katB* mRNA are shown in red and blue boxes, respectively. The percentage of sequence identity identified in the 5′ translated region between nucleotides 62 and 79 of the A1501 *katB* mRNA and homologous sequences in the corresponding regions of *katB* mRNAs from other P. stutzeri strains is shown in parentheses. The match level of base-pairing sequences between *katB* mRNA and two base-pairing sites of NfiS is indicated. (C) Schematic diagrams of the truncated *nfiS* constructs and the resulting survival rate. The survival rate is expressed as in Fig. 2.

Conservation of the structural genes for NfiS in P. stutzeri strains was previously reported (28). Indeed, *katB* genes were also conserved in *P stutzeri* strains or other *Pseudomonas* species, exhibiting a relatively high identity that ranged from 98% to 79% (see Table S1 and Fig. S4 in the supplemental material). These findings are consistent with the conservation of the two base-pairing sites identified in stem-loops of NfiS ncRNAs (Fig. 4A). The match levels between NfiS and the *katB* mRNA ranged from 67% to 89% for site 1 and from 69% to 77% for site 2, and the complementary sequences from *katB* mRNAs were also conserved, with identities of A1501 *katB* ranging from 89% to 100% (Fig. 4B). These results indicate that the two base-pairing sites of NfiS for *katB* mRNA are structurally conserved in P. stutzeri strains.

P. stutzeri ATCC 17588 is a nondiazotrophic strain. We have shown previously that the *nfiS* gene of ATCC 17588 (*nfiS*-ATCC) could restore the oxidative stress-resistant phenotype of the A1501 *nfiS* mutant, but it could not restore nitrogenase activity (28). The identity between the nucleotide sequences of *nfiS*-ATCC and *nfiS*-A1501 is only 79%, while the identities for complementary sequences between NfiS-ATCC and A1501 *katB* mRNA are 83% for site 1 and 77% for site 2. This finding raised the question as to whether the two sites of NfiS-ATCC are functionally conserved among P. stutzeri strains, particularly between ATCC 17588 and A1501. To this end, we constructed two complementation plasmids: pLA*nfiS*-ATCC-tru1, expressing a truncated *nfiS*-ATCC with base-pairing site 1, and pLA*nfiS*-ATCC-tru2, expressing a truncated *nfiS* base-pairing site 2. These two plasmids were then introduced into A1701 (the Δ*nfiS* strain) to yield A1725 and A1724. We observed that both WT and truncated *nfiS*-ATCC restored partial resistance compared with *nfiS*-A1501 (Fig. 4C), strongly suggesting that two the base-pairing sites of NfiS for *katB* mRNA are structurally and functionally conserved in P. stutzeri strains.

### *katB* is involved in the oxidative stress response—probably in an *oxyR*-dependent manner.

**FIG 5:**
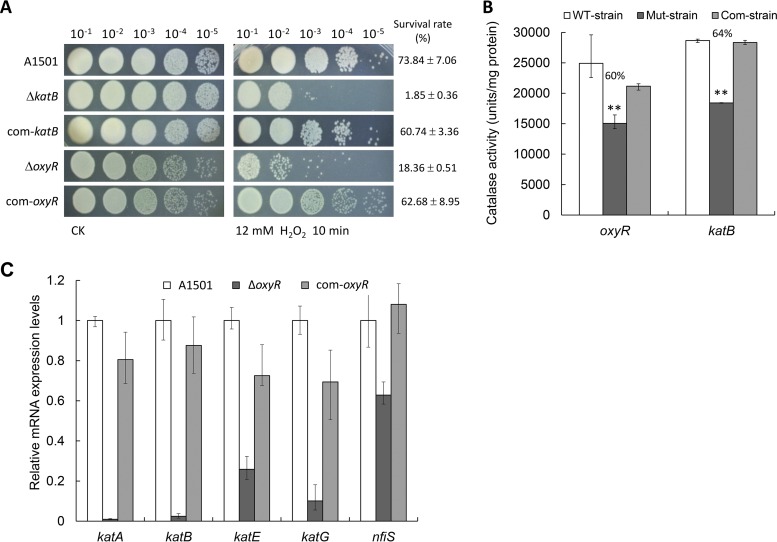
*katB* is activated by OxyR in response to H_2_O_2_ stress. (A) Survival phenotype plate assay of the WT A1501, the A1401 (Δ*katB*) and A1921 (Δ*oxyR*) mutants, and the A1406 (com-*katB*) and A1926 (com-*oxyR*) complemented strains after treatment with 12 mM H_2_O_2_ for 10 min. (B) Effect of *oxyR* or *katB* mutation on total catalase activity. The statistical significance of the difference was confirmed by *t* tests (**, *P* < 0.01). (C) Effect of an *oxyR* mutation on the expression of H_2_O_2_-inducible catalase genes and *nfiS*.

In model systems, such as P. aeruginosa (5) and Pseudomonas putida (29), *katB* expression is induced in response to increased H_2_O_2_ levels by activation of the transcriptional regulator OxyR, and KatB is known as the most pivotal enzyme for detoxifying exogenous H_2_O_2_. In line with this, we predicted that in addition to posttranscriptional regulation by NfiS, the *katB* expression in A1501 was also induced and activated by OxyR at a transcriptional level in response to H_2_O_2_. To conﬁrm this, we constructed two single-gene mutants, insertion mutants (A1401 and A1921) lacking either *katB* or *oxyR*, and their complemented strains (A1406 and A1926) expressing the WT *katB* and *oxyR* genes, respectively, and monitored their oxidative stress resistances. As shown in Fig. 5A, both the *katB* mutant (A1401) and *oxyR* mutant (A1921) were more sensitive than WT strain A1501 following exposure to 12 mM H_2_O_2_. Consistent with their increased sensitivity to oxidative stress, both mutants displayed decreased catalase activities compared to the WT strain (Fig. 5B), indicating their involvement in protecting against oxidative stress. In addition, further analysis of the A1501 *katB* promoter region led to the identification of a putative OxyR binding site (see Fig. S5 in the supplemental material); the sequence consisted of four tetranucleotide elements spaced at 7-bp intervals, similar to those experimentally characterized for the OxyR-binding sequences in P. aeruginosa and P. putida (5, 29) and to the consensus sequence determined in E. coli (30), strongly suggesting a putative OxyR-dependent activation of *katB* expression. This possibility was checked by measuring the expression levels of H_2_O_2_-inducible catalase genes under the *oxyR* mutation background, which show that the *oxyR* mutation resulted in a significant decrease in the expression of H_2_O_2_-inducible catalase genes, especially *katB*, whose expression was almost completely suppressed in the *oxyR* mutant (Fig. 5C). Complementation assays with plasmids carrying either WT *oxyR* or WT *katB* genes confirmed the role of *oxyR* and *katB* in the oxidative stress response. These findings suggest that *katB* expression is dependent on OxyR and is essential for optimal oxidative stress resistance.

### KatB is required for optimal nitrogenase activity.

**FIG 6:**
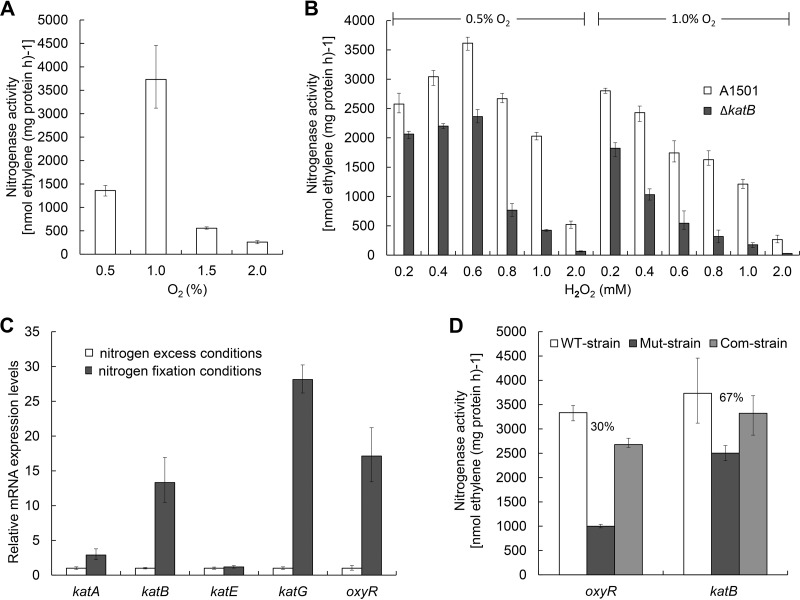
*katB* and OxyR are required for optimal nitrogenase activities. (A) Nitrogenase activity of A1501 at different O_2_ concentrations. (B) Nitrogenase activity of the WT A1501 and the *katB* mutant strain A1401 grown at 1 or 0.5% O_2_ concentrations after treatment with different H_2_O_2_ concentrations. (C) Expression of H_2_O_2_-inducible catalase genes under nitrogen fixation conditions. (D) Effect of *oxyR* or *katB* mutation on nitrogenase activity.

In biological systems, H_2_O_2_ is a key endogenous reactive oxygen species (ROS) from metabolic processes or exogenous ROS from the environment. H_2_O_2_ can be detoxified by catalases, leaving O_2_ as a by-product. O_2_ supports sufficient production of ATP for nitrogenase but can also rapidly limit the activity and repress the synthesis of this enzyme. As yet, the combined effect of O_2_ and H_2_O_2_ on nitrogen fixation has not been analyzed. To this end, the nitrogenase activity of A1501 was examined at different O_2_ (initial concentrations from 0.5% up to 2.0%) and H_2_O_2_ (initial concentrations from 0.2 mM up to 2.0 mM) concentrations. As shown in Fig. 6A, 1.0% is the optimal concentration of O_2_ for optimal nitrogenase activity of A1501, whereas lower or higher concentrations of O_2_ resulted in partial or total loss of activity. At this optimal concentration of O_2_ (1.0%), increasing concentrations of H_2_O_2_ from 0.2 mM to 2.0 mM led to a continued decrease in nitrogenase activity, which was completely inhibited by 2.0 mM H_2_O_2_ (Fig. 6B). In contrast, at a suboptimal O_2_ concentration of 0.5%, an increase in nitrogenase activity of the wild-type strain was observed in the presence of H_2_O_2_ up to 0.6 mM. As was also observed for the wild-type strain A1501, a relatively low level of H_2_O_2_ stress can lead to an increase of nitrogenase activity in the *katB* mutant under O_2_-insufficient conditions (Fig. 6B). We thus presumed that H_2_O_2_ at low concentrations was detoxified by KatB, leaving O_2_ as a by-product to support nitrogen fixation under O_2_-insufficient conditions.

In addition, it is apparent that the nitrogenase activity of the *katB* mutant was more susceptible to increasing H_2_O_2_ concentrations than the wild-type strain. Especially under O_2_-sufficient conditions, the absence of *katB* caused a more significant decrease in nitrogenase activity (Fig. 6B). Consistent with observations mentioned above, the H_2_O_2_-inducible catalase gene, *katB*, and its regulator gene, *oxyR*, were upregulated by approximately 13-fold and 17-fold, respectively, under nitrogen fixation conditions (Fig. 6C). We also determined the nitrogenase activities of A1501, the two mutants, and the corresponding complemented strains. The mutation of the *oxyR* or *katB* genes led to a decrease in nitrogenase activity by approximately 70 or 33% compared to the WT strain, respectively (Fig. 6D). These results suggest that KatB is required for optimal nitrogenase activity, especially at high H_2_O_2_ concentrations and under O_2_-sufficient conditions. We hypothesized that in A1501, nitrogen-fixing conditions led to a significant increase of KatB synthesis (and hence activity), which may be a possible defensive mechanism of nitrogenase activity against oxidative damage.

## DISCUSSION

P. stutzeri A1501 is a root-associated nitrogen-fixing bacterium that can colonize the root surface and invade the root tissues of the host plant. During this process, oxygen appears to be a key regulatory signal controlling the nitrogen fixation process through complex regulatory networks. As shown by its importance in other well-studied *Pseudomonas* strains, ncRNA-mediated regulation is a key component of stress adaptation and gene regulation in nitrogen-fixing P. stutzeri strains. The data presented in this article reveal that NfiS, a P. stutzeri-specific ncRNA, is a multifunctional regulatory RNA that acts by RNA-RNA interactions with two different mRNAs, *nifK* and *katB*. We previously showed that NfiS was recruited by *nifK* mRNA as a novel activator to optimize the nitrogen fixation process in response to specific environmental cues (28). Indeed, the *nfiS* gene was expressed under nitrogen-fixing conditions from an RpoN-dependent promoter, and its expression was strongly impaired in strains carrying mutations in genes controlling the nitrogen fixation process—*rpoN*, *ntrC*, and *nifA*—as well as in an *rpoS*-deficient background (28). We thus proposed a new regulatory mechanism coupling the oxidative stress response and optimal nitrogen fixation, in which NfiS directly pairs with the mRNAs of both a catalase gene, *katB*, and a nitrogenase gene, *nifK*, thereby enhancing the synthesis and activities of both catalase and nitrogenase. The fact that NfiS binding site 1 for *katB* mRNA overlaps the binding site for *nifK* mRNA is puzzling. However, binding to either mRNA may depend on physiological conditions. At oxygen concentrations not compatible with nitrogen fixation, NfiS may control *katB* mRNA using binding sites 1 and 2, while site 1 would be available for binding *nifK* mRNA when oxygen concentrations are compatible with nitrogen fixation. Furthermore, secondary structure analysis of the *katB* and *nifK* mRNAs predicted the formation of a hairpin structure in their coding region downstream of the start codon. Such a hairpin structure is generally believed to affect translation efficiency. These findings suggest that NfiS acts as a pleiotropic riboregulator that integrates adaptation to oxidative stress with other cellular metabolisms and helps to protect nitrogen-fixing cells against oxidative damage.

The genus *Pseudomonas* is one of the most diverse and ecologically significant groups of bacteria on the planet (31). P. stutzeri is a remarkable member of this genus, with exceptional physiological capacities. The diversity within the species is not limited to physiological traits and is also reflected at the genetic level. Catalases are ubiquitous enzymes that detoxify H_2_O_2_ and have been extensively characterized in P. aeruginosa, a species closely related to nitrogen-fixing P. stutzeri (26). P. aeruginosa produces three catalases (KatA, KatB, and KatE) and exhibits a high catalase-specific activity (32, 33). The *katB* gene encoding an H_2_O_2_-inducible catalase is essential for optimal resistance to H_2_O_2_ (34). Multiple catalases are also encoded in the P. stutzeri genome, but the role of each catalase in the response to oxidative stress is still unknown. In P. stutzeri A1501, *katB* inactivation significantly decreased oxidative stress resistance as well as catalase activities. Similar results were observed in P. aeruginosa, suggesting that KatB is the most pivotal enzyme for detoxifying exogenous H_2_O_2_. However, different systems are involved in the regulation of the *katB* gene in A1501 and other *Pseudomonas* strains. To our knowledge, NfiS is the first described case of a bacterial small RNA involved in the regulation of the oxidative stress response via a direct base-pairing interaction with *katB* mRNA. Although NfiS is P. stutzeri specific, the complementary sequences of *katB* mRNA with NfiS are highly conserved and widely distributed not only in P. stutzeri but also in other *Pseudomonas* strains. Based on these data, we propose that the involvement of ncRNAs in the posttranscriptional regulation of the *katB* gene, although not identical to that of NfiS, might be a conserved and widespread mechanism among *Pseudomonas* spp., independent of their ecological features.

Oxidative stress is one of the major challenges for nitrogen-fixing bacteria in almost all environments. Most nitrogen-fixing bacteria are unable to fix nitrogen at high oxygen tensions due to inactivation of nitrogenase. An unusual case is Azotobacter vinelandii, a soil bacterium that can fix nitrogen under aerobic conditions while simultaneously protecting its nitrogenase from oxygen damage (35). This bacterium is equipped with particular physiological mechanisms, such as the autoprotection of nitrogenase with the involvement of catalase (36), the respiratory protection of terminal oxidases (35), the oxygen barrier of the alginate capsule (37), and conformational protection of nitrogenase due to association with an FeSII protein, named “Shethna protein” (38, 39). In addition, the roles of the A. vinelandii RpoS, Kat1, and RgsA ncRNAs in the survival of oxidative stress have been well documented (40, 41). Another striking example is rhizobial infection, during which reactive oxygen species such as H_2_O_2_ are transiently produced. The symbiotic nitrogen-fixing model bacterium Sinorhizobium meliloti possesses three distinct catalases (KatB, KatA, and KatC) to cope with oxidative stress (42). KatB activity was detected throughout the growth of S. meliloti in minimal medium and represented approximately 90% of the total catalase activity, suggesting an important role of this bifunctional catalase in free-living bacteria (43). In addition, the *katB katC* double mutant displayed reduced nitrogen fixation and abnormal infection, indicating that these two catalases are essential for the establishment of symbiosis (44).

Oxygen control, which is tightly controlled in response to the external oxygen concentration, is exerted first at the transcriptional level of the *nif* operons and then at the level of the nitrogenase activity. At the transcriptional level, oxygen repressed nitrogenase synthesis much more rapidly than ammonia did in *Azotobacter* (45). In most *Proteobacteria*, including P. stutzeri A1501, NifA is the transcriptional activator of other *nif* operons (46–50). In K. pneumoniae strains that can fix nitrogen anaerobically, NifL responds to oxygen and prevents NifA-mediated activation of *nif* gene expression (46, 47). In P. stutzeri, whose optimal nitrogenase activity was observed at an oxygen concentration of 1%, the expression of *nifLA* was also controlled by oxygen (22, 49). Transcription of *nif* genes from Rhodobacter capsulatus, a bacterium that does not contain an *nifL* gene, is inhibited by oxygen, probably through direct inactivation of NifA (50). At the next level, oxygen causes rapid and irreversible damage to nitrogenase enzymes (51). In *Azotobacter*, the FeSII ferredoxin “Shethna protein” plays a protective role against oxygen damage by forming a reversibly inactive complex with nitrogenase. This mechanism is also likely present in Klebsiella pneumoniae and *Gluconacetobacter* (52) but may be absent in P. stutzeri A1501 because the gene encoding an FeSII protein is absent from the A1501 genome.

**FIG 7:**
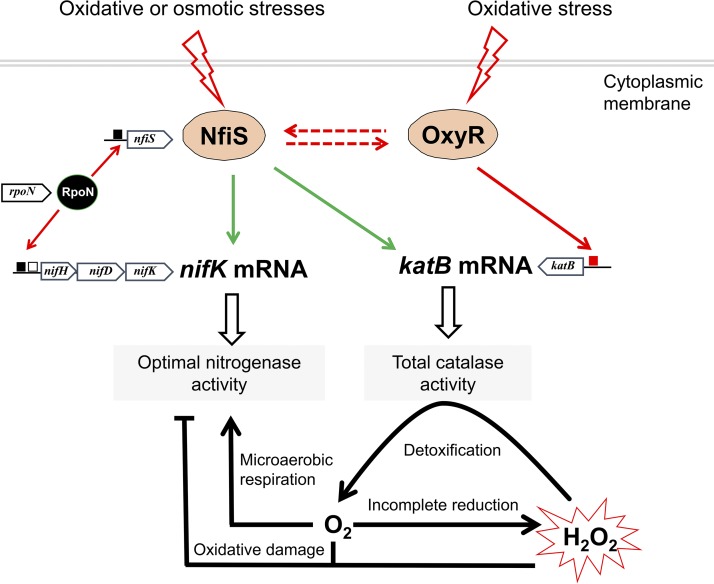
Model for the NfiS-mediated regulatory network and oxidative stress response in P. stutzeri A1501, indicating data supported by our own experiments in this study and the previous study (40). NfiS acts as a pleiotropic riboregulator to optimize catalase and nitrogenase activities via direct base pairing with *katB* mRNA and *nifK* mRNA or via the indirect transcriptional activation of OxyR. This global regulation integrates adaptation to oxidative stress with other cellular metabolic processes and helps to protect nitrogen-fixing cells against oxidative damage. Under O_2_-insufficient conditions, H_2_O_2_ at low concentrations is detoxified by H_2_O_2_-inducible catalases, leaving O_2_ as a by-product to support nitrogen fixation. The black box represents the putative RpoN-dependent promoters, the red box represents the putative OxyR-dependent promoter, and the white box represents the upstream activator NifA sequences. Solid red or green arrows indicate transcriptional regulation and posttranscriptional regulation, respectively, and dashed lines represent unknown mechanisms. For more details, see the Discussion.

Together, these findings suggest that oxygen supports sufficient production of ATP for nitrogenase but can also rapidly limit the activity and repress the synthesis of this enzyme. Therefore, nitrogen-fixing bacteria must develop dynamic and intricate strategies to quickly fine-tune gene expression in response to environmental stimuli. In addition to specific regulatory proteins, ncRNAs have been identified as components of regulatory networks and modulate various physiological processes, especially stress adaptation (53). Furthermore, posttranscriptional regulation by ncRNAs allows a cell to respond to the signal in an extremely flexible and sensitive manner (14, 54). Few regulatory RNAs and their targets have been functionally characterized in nitrogen-fixing bacteria. Regulatory ncRNA-mediated responses to environmental stimuli provide an adaptive advantage to nitrogen-fixing bacteria, such as observed for A. vinelandii ArrF (55), S. meliloti EcpR1 (56), P. stutzeri NfiS (28), and Methanosarcina mazei sRNA_154_ (57). A single sRNA often modulates a particular physiological response via multiple target genes and could be used by cells to integrate multiple signals into gene expression programs (15, 58). This study and other preliminary results also suggest that in P. stutzeri A1501, NfiS-mediated processes might work together in a more sophisticated manner than previously believed. The working model for NfiS in P. stutzeri (Fig. 7) tentatively illustrates the regulatory roles of NfiS in integrating adaptation to oxidative stress with other cellular metabolisms and will broaden our knowledge of ncRNA-based regulatory mechanisms in environmental microorganisms. At the posttranscriptional level, NfiS directly pairs with the mRNAs of both the catalase gene *katB* and the nitrogenase gene *nifK*, thus linking total catalase activity and optimal nitrogen fixation. At the transcriptional level of global regulation, NfiS expression is strongly decreased in *rpoN*, *ntrC*, and *nifA*, which supports the former conclusion that *nfiS* is a nitrogen fixation-regulated gene (28); a significant decrease is also observed in the *oxyR* mutant. Thus, NfiS expression depends on OxyR, NifA, NtrC, and RpoN, implying a more complex regulatory circuitry. In addition, NfiS, known to be involved in survival of oxidative stress in other systems, may also play a similar role (28), which remains to be elucidated.

The regulatory mechanisms of the oxidative stress response in P. stutzeri A1501 have not been fully elucidated. We previously showed that NfiS synthesis was significantly induced by sorbitol and that the NfiS mutant strain displayed increased sensitivity to sorbitol, suggesting that NfiS in P. stutzeri is also involved in the regulation of the osmotic stress response (28). Therefore, the possibility that NfiS contains another base-pairing site with target genes involved in osmotic stress response remains to be explored. Future studies should help to understand how P. stutzeri A1501 defends itself against reactive oxygen species generated under various conditions and to determine why the bacterium is highly adaptable to environmental changes.

## MATERIALS AND METHODS

### Strains, plasmids, oligonucleotides, media, and culture conditions.

All the strains and plasmids used and constructed in this study are listed in Table S2 in the supplemental material. P. stutzeri A1501 and mutant derivatives were grown in LB or in minimal lactate medium (medium K [pH 6.80]) at 30°C (22). Spectinomycin (40 μg ml^−1^), tetracycline (10 μg ml^−1^), or kanamycin (35 μg ml^−1^) was added to the medium when needed. For cloning procedures, E. coli TOP10 competent cells (CWBIO) were grown in LB broth or on LB agar plates.

### Construction of *katB* and *oxyR* nonpolar insertion mutants and complementation plasmids.

The *katB* and *oxyR* genes were inactivated by homologous suicide plasmid integration using pK18mob as a vector (59). Oligonucleotide primers were designed to generate internal gene fragments of 145 bp for *katB* and 290 bp for *oxyR* by PCR, enabling the creation of mutations in *katB* and *oxyR* without preventing the transcription of their downstream genes (PST3567 and PST0135, respectively). The amplicons obtained were doubly digested with EcoRI/BamHI and EcoRI/HindIII and then cloned into pK18mob. The resulting plasmids were introduced into A1501 by triparental mating, generating *katB* and *oxyR* nonpolar insertion mutant strains, named A1401 and A1901, respectively (Table S2). Correct recombination was confirmed by PCR, followed by nucleotide sequencing of the amplicons obtained. DNA fragments containing WT genes for *katB* or *oxyR* with their promoter and terminator regions were amplified by PCR to construct complementation plasmids. A 1,800-bp fragment containing *katB* and a 1,453-bp fragment containing *oxyR* were doubly digested with HindIII/BamHI and EcoRI/SalI, respectively, and then ligated into pLAFR3 to yield the complementation plasmids pLA*katB* and pLA*oxyR*. Both plasmids were then introduced into A1401 or A1921 by triparental mating, generating strains A1406 and A1926.

### Construction of complementation plasmids carrying the WT or truncated or mutated *nfiS* genes.

Two plasmids carrying the WT *nfiS* gene from two different P. stutzeri strains and seven plasmids carrying truncated or mutated *nfiS* genes were constructed using the broad host plasmid pLAFR3 as a vector (60) to complement the constructed A1701 deletion strain. Nine *nfiS* DNA fragments with their promoter and terminator regions were synthesized by BGI Co., Ltd. Restriction enzyme sites (HindIII and BamHI) were incorporated into synthesized fragments, which were subsequently cloned into the pLAFR3 vector, resulting in the following plasmids: pLA*nfiS*-A1501, expressing an A1501 WT *nfiS*; pLA*nfiS*-tru1, expressing a truncated *nfiS* carrying the first 54 nucleotides with base-pairing site 1; pLA*nfiS*-tru2, expressing a truncated *nfiS* carrying the 200 remaining nucleotides with base-pairing site 2; pLA*nfiS*-mS1, expressing a mutated *nfiS* lacking base-pairing site 1; pLA*nfiS*-mS2, expressing a mutated *nfiS* lacking base-pairing site 2; pLA*nfiS*-dmS12, expressing a mutated *nfiS* with a double mutation of the two sites; pLA*nfiS*-ATCC, expressing an ATCC 17588 WT *nfiS*; pLA*nfiS*-ATCC-tru1, expressing an ATCC 17588 truncated *nfiS* carrying the first 44 nucleotides with site 1; and pLA*nfiS*-ATCC-tru2, expressing an ATCC 17588 truncated *nfiS* carrying the 200 remaining nucleotides with site 2 (Table S2).

### Cell survival under H_2_O_2_ stress.

The cell susceptibility of P. stutzeri A1501 and its derivatives to H_2_O_2_ was assayed as previously described (61). Strains were grown overnight in LB broth at 30°C and were transferred into fresh LB broth up to an optical density at 600 nm (OD_600_) of 0.6. Then, 12 mM H_2_O_2_ was added to the medium, and the cultures were incubated at 30°C and 220 rpm for 10 min. Serial 10-fold dilutions of OD-standardized cultures were spotted on LB plates. Plates were incubated at 30°C for 24 h prior to colony enumeration. The survival rate was expressed as the percentage of the number of colonies in the treated samples compared with that in the untreated A1501 sample used as a control.

### qRT-PCR.

Total RNA was isolated with an innuPREP RNA minikit (Analytik Jena) and was reverse-transcribed into cDNA, which was diluted to 100 ng μl^−1^. Quantitative real-time PCR (qRT-PCR) assays were performed using total RNA preparations obtained from three independent cultures (three biological replicates). The gene-specific primers listed in Table S3 in the supplemental material were designed based on the full genome sequence of P. stutzeri A1501, and the 16S rRNA gene was used as the endogenous reference gene to normalize the expression of target genes in each cDNA template. The relative mRNA concentration was calculated by the comparative threshold cycle (2^−^*^ΔΔCT^*) method. The target gene copy numbers were determined in triplicate using the 7500 real-time PCR system and ChamQ SYBR qPCR master mix. All procedures were carried out according to the manufacturers’ recommendations. Data were analyzed using ABI Prism 7500 sequence detection system software (Applied Biosystems).

### Computational target prediction.

The sTarPicker prediction method (62) was used to predict interactions between NfiS and all annotated open reading frames (ORFs) of A1501. The interaction region on mRNA levels was defined as 100 bp up- and downstream of the start codon.

### Microscale thermophoresis measurements.

MST experiments were performed according to Zhan et al. (28). A modified overlap extension PCR method was used to amplify the full-length wild-type NfiS and its mutated products using mutagenic primers (Table S3). A set of full-length ncRNAs (N-NfiS probes) for MST was then transcribed by GenePharma using a MAXIscript kit (Thermo Fisher) from PCR-generated templates (see Table S4 in the supplemental material). In addition, another set of Cy3-labeled 70-nt single-stranded RNA (ssRNA) oligonucleotides containing WT or mutated base-pairing regions of *katB* mRNA (N-*katB* competitors) were synthesized by GenePharma Company (Table S4). Four microliters of sample containing 200 nM labeled probe and increasing concentrations of a nonlabeled competitor (from 5 nM to 150 μM) were loaded on standard treated silicon capillaries (Monolith NT.115 series capillaries; catalog no. MO-K002). The measurements were carried out using a Monolith NT.115 instrument (NanoTemper Technologies GmbH) at 26°C in diethyl pyrocarbonate (DEPC)-treated water with 40% excitation power and medium MST-Power. The dissociation constants (*K_d_*) were calculated as described previously (63). Data analyses were performed using Nanotemper Analysis software (NanoTemper Technologies).

### Nitrogenase activity assays.

Nitrogenase activity was determined by the acetylene reduction test (64) using a derepression protocol (22), as follows. Cells from an overnight culture in LB medium were centrifuged and resuspended in a 60-ml flask containing 10 ml of N-free minimal medium K at an OD_600_ of 0.1. The suspension was incubated at 30°C, with vigorous shaking, under an argon atmosphere containing 1.0% oxygen and 10% acetylene. Gas samples (0.25 ml) were taken at regular intervals to determine the amount of ethylene produced. Samples were analyzed on a polydivinylbenzene porous bead GDX-502 column using a gas chromatograph SP-2100 fitted with a flame ionization detector (Beijing Beifen-Ruili Analytical Instrument Co., Ltd.). The content of ethylene in the gas samples was determined by reference to an ethylene standard. Each experiment was repeated at least three times. Protein concentrations were determined by the Bio-Rad protein assay. For the nitrogenase activity of A1501 and *katB* mutant A1401, different concentrations of H_2_O_2_ (0.2, 0.4, 0.6, 0.8, 1.0, and 2.0 mM) were added to the samples immediately after inoculation of the bacterial samples, and then the flasks were incubated under an argon atmosphere containing 10% acetylene and 0.5 or 1.0% oxygen.

### Catalase activity assays.

The total catalase activity of the samples was determined using a catalase assay kit (Beyotime Institute of Biotechnology, Jiangsu, China) based on the protocols provided by the manufacturer. The catalase activity assays were carried out at 37°C in flasks in the presence of H_2_O_2_ (concentration of 12 mM). Cells were washed twice with phosphate-buffered saline (PBS) and collected, lysed by cell lysis buffer, and centrifuged at 10,000 × *g* for 15 min at 4°C. Then the clear supernatants were collected, and the assay was performed immediately. The H_2_O_2_ concentration of this kit can be calculated by *C* (mM) = 22.94 × *A*_240_ according to the protocol, where *C* is the concentration of H_2_O_2_ and *A*_240_ is the absorbance of the reaction solution at 240 nm. Samples were prepared and added to the 96-well plate. Then the mixtures were incubated at 25°C for 15 min at least (but no more than 45 min), and the absorbance was finally determined at 520 nm. The experiments were performed three times independently, and the catalase levels were normalized against total protein levels.

### Stability measurements of *katB* mRNA.

For determination of the half-life of *katB* mRNA under oxidative stress conditions, 12 mM H_2_O_2_ was added to LB medium-grown cultures (OD_600_ of 0.6) as indicated above. Rifampin (40 mg/ml) was added immediately after H_2_O_2_ shock. Then 2-ml samples were collected at different times (0, 1, 3, 5, and 7 min). RNAlater (Sigma) was added to 2 volumes of RNAprotect. After incubation at room temperature for 5 min, samples were centrifuged for 2 min at 12,000 rpm, and pellets were quickly frozen in liquid nitrogen and stored at −80°C until use. Total RNA was isolated using an innuPREP RNA minikit (Analytik Jena), and cDNA was synthesized from total RNA using a PrimeScript RT reagent kit with gDNA Eraser (Perfect Real Time) and was used to estimate mRNA levels by qRT-PCR. The primers used to measure the half-life of *katB* transcripts are listed in Table S3, and qRT-PCR was performed as described above. The relative mRNA concentration was calculated by the comparative threshold cycle (2^−^*^ΔΔCT^*) method with 16S rRNA as the endogenous reference. Gene expression for each time point was normalized to the endogenous reference. Data are presented as the percentage of *katB* mRNA levels relative to the amount of these mRNAs at time point zero.

## Supplementary Material

Supplemental file 1
