# Supplementary material for: The Pseudomonas stutzeri-Specific Regulatory Noncoding RNA NfiS Targets katB mRNA Encoding a Catalase Essential for Optimal Oxidative Resistance and Nitrogenase Activity
Source: J Bacteriol. 2019 Sep 6;201(19):e00334-19. doi: 10.1128/JB.00334-19 (PMC6755748; doi:10.1128/JB.00334-19)
Supplement: Supplemental file 1 [file JB.00334-19-s0001.pdf]

Table S1 Nucleotide sequence identity (%) of the *katA*, *katB*, *katE*, *katG*, and *oxyR* genes in different *Pseudomonas* strains relative to those of *P. stutzeri* A1501.

	P. aeruginosa PAO1	*P. fluorescens* SBW25	*P. putida* 1A00316	*P. syringae* B301D	*P. stutzeri* RCH2	*P. stutzeri* DSM4166	*P. stutzeri* DW2-1	*P. stutzeri* ATCC17588
katA	69%	76%	77%	77%	68%	98%	69%	98%
katB	82%	79%	83%	78%	94%	98%	98%	98%
katE	76%	74%	75%	78%	92%	99%	92%	99%
katG	—	72%	70%	69%	85%	99%	72%	99%
oxyR	89%	86%	85%	82%	93%	99%	94%	99%

Table S2 Strains and plasmids used in this study.

Strain or plasmid	Relevant characteristics	Source
***P. stutzeri* strains**		
A1501	WT, Chinese Culture Collection: CGMCC 0351	(1)
A1701 (Δ*nfiS*)	*nfiS* deletion mutant, $Spec^r$	(2)
A1706	A1701 containing pLA*nfiS*-A1501, $Spec^r$ and Tc^r	(2)
A1717	A1701 containing pLA*nfiS*-mS1, $Spec^r$ and Tc^r	This study
A1718	A1701 containing pLA*nfiS*-mS2, $Spec^r$ and Tc^r	This study
A1719	A1701 containing pLA*nfiS*-dmS12, $Spec^r$ and Tc^r	This study
A1715	A1701 containing pLA*nfiS*-tru1, $Spec^r$ and Tc^r	This study
A1714	A1701 containing pLA*nfiS*-tru2, $Spec^r$ and Tc^r	This study
A1708	A1701 containing pLA*nfiS*-ATCC, $Spec^r$ and Tc^r	(2)
A1725	A1701 containing pLA*nfiS*-ATCC-tru1, $Spec^r$ and Tc^r	This study
A1724	A1701 containing pLA*nfiS*-ATCC-tru2, $Spec^r$ and Tc^r	This study

A1401 (Δ*katB*)	*katB* insertion mutant, Km^r	This study
A1406	A1401 containing pLA*katB*, Km^r and Tc^r	This study
A1921(Δ*oxyR*)	*oxyR* insertion mutant, Km^r	This study
A1926	A1921 containing pLA*oxyR*, Km^r and Tc^r	This study
***E. coli* strains**		
E. coli DH5α	Competent cell for cloning	CWBIO Company
**Plasmids**		
pLAFR 3	Mobilizable vector, Tc^r	(3)
pRK2013	Helper plasmid for conjugation into *P. stutzeri* A1501, Km^r	(4)
pK18mob	Mobilizable plasmid containing an *Escherichia coli* origin of replication, Km^r	(5)
pLA*nfiS*-A1501	pLAFR 3 derivative carrying the A1501 WT *nfiS* gene under the control of its endogenous promoter, Tc^r	(2)
pLA*nfiS*-ATCC	pLAFR 3 derivative carrying the ATCC17588 WT *nfiS* gene under the control of its endogenous	(2)

	promoter, Tc^r	
pLA*nfiS*-tru1	pLAFR 3 derivative carrying the A1501 truncated *nfiS* gene containing only base-pairing site 1, Tc^r	This study
pLA*nfiS*-tru2	pLAFR 3 derivative carrying the A1501 truncated *nfiS* gene containing only base-pairing site 2, Tc^r	This study
pLA*nfiS*-ATCC-tru1	pLAFR 3 derivative carrying the ATCC17588 truncated *nfiS* gene containing only base-pairing site 1, Tc^r	This study
pLA*nfiS*-ATCC-tru2	pLAFR 3 derivative carrying the ATCC17588 truncated *nfiS* gene containing only base-pairing site 2, Tc^r	This study
pLA*nfiS*-mS1	pLAFR 3 derivative carrying the A1501 mutated *nfiS* gene with a substitution of 16 nucleotides in the base-pairing site 1 region, Tc^r	This study
pLA*nfiS*-mS2	pLAFR 3 derivative carrying the A1501 mutated *nfiS* gene with a substitution of 10 nucleotides in the base-pairing site 2 region, Tc^r	This study
pLA*nfiS*-dmS12	pLAFR 3 derivative carrying the A1501 mutated *nfiS* gene with double mutations of two sites, Tc^r	This study

pK18mob-*katB*	pK18mob derivative carrying the A1501 *katB* insertion fragment, Km^r	This study
pLA*katB*	pLAFR 3 derivative carrying the A1501 WT *katB* gene under the control of its endogenous promoter, Tc^r	This study
pK18mob-*oxyR*	pK18mob derivative carrying the A1501 *oxyR* insertion fragment, Km^r	This study
pLA*oxyR*	pLAFR 3 derivative carrying the A1501 WT *oxyR* gene under the control of its endogenous promoter, Tc^r	This study

Table S3 Primers used in this study.

Primer[a]	Sequence (5'-3')[b]	Purpose
qRT-16S F	CCTACGGGAGGCAGCAG	
qRT-16S R	ATTACCGCGGCTGCTGG	
qRT-katA F	ATGGACCAATCTGAAGAGCC	
qRT-katA R	CGTGCATGAACCGATAACC	
qRT-katB F	CTTCTTGCTGAACGAGCGATAC	
qRT-katB R	TTCTCCTACGCCGATACCCA	
qRT-katE F	GCTGGACCCGACCAAAAT	
qRT-katE R	CGGACGGTTGATCGGAAT	
qRT-katG F	TTCCGCAACTACTACCACGAG	
qRT-katG R	TGTCCAGCAGGTTGACGAAG	
qRT-sodB F	AAGGAAGAGTTCACCAAGACCG	
qRT-sodB R	ACGAAGTCCCAGTTCACCAG	

qRT-sodC F	CACGGCTTTCATATCCACG	
qRT-sodC R	CACGGCTTTCATATCCACG	
qRT-ahpC F	GTCTTCTCGCCCTCTTTCCA	
qRT-ahpC R	AGATCGCTCGTGACGTGTCC	
qRT-nfiS F	ACTGCTGATCCATCTGCTGAG	
qRT-nfiS R	CTGCATCAGCGGGCAATG	
Insert-katB F	TATGACCATGATTACGAATTCCGGCGGTCTGGGAATTCT	pK18mob-*katB* construct
Insert-katB R	CAGGTCGACTCTAGAGGATCCTCCCGTATGACCTTTGGCTC	
Insert-oxyR F	GAATTCTTGTCGTTGAGCATCTCCGC	pK18mob-*oxyR* construct
Insert-oxyR R	AAGCTTCTCAAGGTCGGTGCCATCTA	
YZ-katB F	GCCGATTCATTAATGCAGCTGG	Validation of pK18mob-*katB*
YZ-katB R	TCCCGTATGACCTTTGGCTC	
YZ-oxyR F	GAATTCTTGTCGTTGAGCATCTCCGC	Validation of pK18mob-*oxyR*
YZ-oxyR R	CATTCAGGCTGCGCAACTGT	

Con-katB F	GCCGATTCATTAATGCAGCTGG	Validation of A1401 (Δ*katB*) by PCR
Con-katB R	ATTCAATTGGGGCATCTTCGG	
Con-oxyR F	TTTCCAGCGAGCTGGATT	Validation of A1921 (Δ*oxyR*) by PCR
Con-oxyR R	GTGTACTGATCTTCGAAC	
Com-katB F	ACGACGGCCAGTGCCAAGCTTGAAACAACCGGCACCCAT	pLA*katB* construct
Com-katB R	TAATGCTTAAGGGCCGGATCCCGGTGGCGAATGTCTCATC	
Com-oxyR F	GAATTCCGATGCCTTCGATCACCG	pLA*oxyR* construct
Com-oxyR R	GTCGACCATAGACCAGATAATCCACG	
Com-YZ-katB F	TTGTAAAACGACGGCCAGTG	Validation of A1406 by PCR
Com-YZ-katB R	TCCCGTATGACCTTTGGCTC	
Com-YZ-oxyR F	TTGTAAAACGACGGCCAGTG	Validation of A1926 by PCR
Com-YZ-oxyR R	GTCGACCATAGACCAGATAATCCACG	
Con-A1501-nfiS F	CTCTACGGTTCGCTGCGTA	Validation of different lengths of *nfiS*-A1501 derivatives

Con-A1501-nfiS R	GTCAGGCGAGGGGAGAAG	
Con-17588-nfiS F	AGCCGGATGAGAATCGAAG	Validation of different lengths of *nfiS*-ATCC derivatives
Con-17588-nfiS R	AGGTTTCCTTGTGCAACAGG	
Y5598-1	AGCCCGGGCTCGAGAGGAAATTAATACGACTCACTATAGGGAGAATCGCCGATGACACGCCGCC	Template amplification of full-length NfiS-wt
Y5598-2	GCTCAGCAGATGGATCAGCAGTGCCAGGCTGGCGAGCCAGGCGGGGAGGCGGCGGCGTGTCATCG	
Y5598-3	GCTGATCCATCTGCTGAGCATGCCGCTGTCTGGCCTGTTGCCGGCGGATGCCAAGCGGCTGCTCG	
Y5598-4	ATGCGCCTGCTGCACCTGCGCCTGCATCAGCGGGCAATGCCCACTCCAGCCGAGCAGCCGCTTGG	
Y5598-5	GTGCAGCAGGCGCATGCGCTGCACCCGCCGGCAGTCCATGGCGAGCATGATTCGGGCACCCATGG	

Y5598-6	ATCCAAGCTTCGGCACAGCAGCAGCAAGGCGGCATCCCGCCGTGCG CGCCATGGGTGCCCGAATC	
Y5599-1	AGCCCGGGCTCGAGAGGAAATTAATACGACTCACTATAGGGAGAAT CGCCGATGACACGCCGCC	Template amplification of full-length NfiS1-mut6
Y5599-2	GCTCAGCAGATGGATCAGCAGTGCCAGGCTGGCGAGCCAGGCGGGG AGGCGGCGGCGTGTCATCG	
Y5599-3	GCTGATCCATCTGCTGAGCATGCCGCTGTCTGGCCTGTTGCCGGCGG ATGCCAAGCGGCTGCTCG	
Y5599-4	ATGCGCCTGCTGCACCTGCGCCTGCATCAGCGGGCAATGCCCACTCC AGCCGAGCAGCCGCTTGG	
Y5599-5	GTGCAGCAGGCGCATGCGCTGCACCCGCCGGCAGTCCATGGCGAGC ATGATTCGGGCACCCATGG	
Y5599-6	TccAAGCTTCGGCAGTCGTCCAGCAAGGCGGCATCCCGCCGTGCGCGc catgggtgcccgaatca	

Y5600-1	AGCCCGGGCTCGAGAGGAAATTAATACGACTCACTATAGGGAGAATCGCCGATGACACGCCGCC	Template amplification of full-length NfiS2-mut4
Y5600-2	GCTCAGCAGATGGATCAGCAGTGCCAGGCTGGCGAGCCAGGCGGGGAGGCGGCGGCGTGTCATCG	
Y5600-3	gatccatctgctgagcaTGCCGCTGTCTGGCCTGTTGCCGGCGGATGCCAAGCGCGACCTCGGct	
Y5600-4	atgcgcctgctgcacCTGCGCCTGCATCAGCGGGCAATGCCCACTCCagCCGAGGTCGCGCTTGG	
Y5600-5	GTGCAGCAGGCGCATGCGCTGCACCCGCCGGCAGTCCATGGCGAGCATGATTCGGGCACCCATGG	
Y5600-6	ATCCAAGCTTCGGCACAGCAGCAGCAAGGCGGCATCCCGCCGTGCGCGCCATGGGTGCCCGAATC	
Y5601-1	AGCCCGGGCTCGAGAGGAAATTAATACGACTCACTATAGGGAGAATCGCCGATGACACGCCGCC	Template amplification of full-length NfiS1-com

Y5601-2	GCTCAGCAGATGGATCAGCAGTGCCAGGCTGGCGAGCCAGGCGGGG AGGCGGCGGCGTGTCATCG	
Y5601-3	GCTGATCCATCTGCTGAGCATGCCGCTGTCTGGCCTGTTGCCGGCGG ATGCCAAGCGGCTGCTCG	
Y5601-4	ATGCGCCTGCTGCACCTGCGCCTGCATCAGCGGGCAATGCCCACTCC AGCCGAGCAGCCGCTTGG	
Y5601-5	GTGCAGCAGGCGCATGCGCTGCACCCGCCGGCAGTCCATGGCGAGC ATGATTCGGGCACCCATGG	
Y5601-6	TccAAGCTTCGGCACAGCAGCAGCAAGGGGCATCCCGCCGTGCGCGcc ATGGGTGCCCGAATCAT	
Y5602-1	AGCCCGGGCTCGAGAGGAAATTAATACGACTCACTATAGGGAGAAT CGCCGATGACACGCCGCC	Template amplification of full-length NfiS2-com
Y5602-2	GCTCAGCAGATGGATCAGCAGTGCCAGGCTGGCGAGCCAGGCGGGG AGGCGGCGGCGTGTCATCG	

Y5602-3	tgatccatctgctgagcaTGCCGCTGTCTGGCCTGTTGCCGGCGGATGCCGCGG CTGCTCGGCTg
Y5602-4	gcatgcgcctgctgcacCTGCGCCTGCATCAGCGGGCAATGCCCACTCcAGCC GAGCAGCCGCGG
Y5602-5	GTGCAGCAGGCGCATGCGCTGCACCCGCCGGCAGTCCATGGCGAGC ATGATTCGGGCACCCATGG
Y5602-6	ATCCAAGCTTCGGCACAGCAGCAGCAAGGCGGCATCCCGCCGTGCG CGCCATGGGTGCCCGAATC

[a]F, forward primer; R, reverse primer.

[b]Restriction sites are underlined.

Table S4 Synthesized ssRNA oligonucleotide derivatives for MST

Name*	Sequence (5'-3') †	Relevant characteristics
N-NfiS-wt	CGGCACAGCAGCAGCAAGGCGGCAUCCCGCCGUGCGCGCCAUGGGU GCCCGAAUCAUGCUCGCCAUGGACUGCCGGCGGGUGCAGCGCAUGC GCCUGCUGCACCUGCGCCUGCAUCAGCGGGCAAUGCCCACUCCAGC CGAGCAGCCGCUUGGCAUCCGCCGGCAACAGGCCAGACAGCGGCAU GCUCAGCAGAUGGAUCAGCAGUGCCAGGCUGGCGAGCCAGGCGGG GAGGCGGCGGCGUGUCAUCGGCGAU	wild type (254 bp), interaction with N-*katB*R-wt
N-NfiS1-com	CGGCACAGCAGCAGCAAGG**A**GGCAUCCCGCCGUGCGCGCCAUGGGU GCCCGAAUCAUGCUCGCCAUGGACUGCCGGCGGGUGCAGCGCAUGC GCCUGCUGCACCUGCGCCUGCAUCAGCGGGCAAUGCCCACUCCAGC CGAGCAGCCGCUUGGCAUCCGCCGGCAACAGGCCAGACAGCGGCAU GCUCAGCAGAUGGAUCAGCAGUGCCAGGCUGGCGAGCCAGGCGGG GAGGCGGCGGCGUGUCAUCGGCGAU	compensatory mutation (254 bp), stronger interaction with N-*katB*R-wt

N-NfiS2-com	CGGCACAGCAGCAGCAAGGCGGCAUCCCGCCGUGCGCGCCAUGGGU<br>GCCCGAAUCAUGCUCGCCAUGGACUGCCGGCGGGUGCAGCGCAUGC<br>GCCUGCUGCACCUGCGCCUGCAUCAGCGGGCAAUGCCCACUCCAGC<br>CGAGCAGCCGC**AA**GGCAUCCGCCGGCAACAGGCCAGACAGCGGCAU<br>GCUCAGCAGAUGGAUCAGCAGUGCCAGGCUGGCGAGCCAGGCGGG<br>GAGGCGGCGGCGUGUCAUCGGCGAU	compensatory mutation (254 bp), stronger interaction with N-*katB*R-wt
N-NfiS1-mut6	CGGCA**GUCGUC**CAGCAAGGCGGCAUCCCGCCGUGCGCGCCAUGGGU<br>GCCCGAAUCAUGCUCGCCAUGGACUGCCGGCGGGUGCAGCGCAUGC<br>GCCUGCUGCACCUGCGCCUGCAUCAGCGGGCAAUGCCCACUCCAGC<br>CGAGCAGCCGCUUGGCAUCCGCCGGCAACAGGCCAGACAGCGGCAU<br>GCUCAGCAGAUGGAUCAGCAGUGCCAGGCUGGCGAGCCAGGCGGG<br>GAGGCGGCGGCGUGUCAUCGGCGAU	mismatch mutation (254 bp), weaker interaction with N-*katB*R-wt
N-NfiS2-mut4	CGGCACAGCAGCAGCAAGGCGGCAUCCCGCCGUGCGCGCCAUGGGU<br>GCCCGAAUCAUGCUCGCCAUGGACUGCCGGCGGGUGCAGCGCAUGC	mismatch mutation (254 bp), weaker interaction with N-*katB*R-wt

	GCCUGCUGCACCUGCGCCUGCAUCAGCGGGCAAUGCCCACUCCAGC	
	CGAG**GUCG**CGCUUGGCAUCCGCCGGCAACAGGCCAGACAGCGGCAU	
	GCUCAGCAGAUGGAUCAGCAGUGCCAGGCUGGCGAGCCAGGCGGG	
	GAGGCGGCGGCGUGUCAUCGGCGAU	
N-*katB*R-wt	CCUUGCGCUGGGCGUUAUUUCCGCGAGCCUCCUUGCGCUGUCUGCC	wild type (70 bp), interaction with N-NfiS-wt
	AAUGCCGCCCCGCUGACCCGCGAC	
N-*katB*R1-com	CCUUGCGCUGGGCGUUAUUUCCGCGAGCC**G**CCUUGCGCUGUCUGCC	compensatory mutation (70 bp), stronger interaction
	AAUGCCGCCCCGCUGACCCGCGAC	with N-NfiS-wt
N-*katB*R2-com	CCUUGCGCUGGGCGUUAUUUCCGCGAGCCUCC**AA**GCGCUGUCUGCC	compensatory mutation (70 bp), stronger interaction
	AAUGCCGCCCCGCUGACCCGCGAC	with N-NfiS-wt
N-*katB*R1-mut6	CCUUGCGCUGGGCGUUAUUUCCGCGAGCCUCCUUGCG**GAC**U**GAC**CC	mismatch mutation (70 bp), weaker interaction with
	AAUGCCGCCCCGCUGACCCGCGAC	N-NfiS-wt
N-*katB*R2-mut4	CCUUGCGCUGGGCGUUAUUUCCGCGAGCCUCCUUGC**CGAC**UCUGCC	mismatch mutation (70 bp), weaker interaction with
	AAUGCCGCCCCGCUGACCCGCGAC	N-NfiS-wt

*N: 30-nt ssRNA oligonucleotide; wt: wild type; mut: mutation; *katB*R: *katB* mRNA

[†]The 18-nt/13-nt sequence of the NfiS stem-loop 1/2 pairing with *katB* mRNA is shown in red. The 18-nt/13-nt sequence of the complementary region at the 5' end of *katB* mRNA is shown in blue. Point mutations introduced into synthesized oligonucleotide derivatives are underlined.

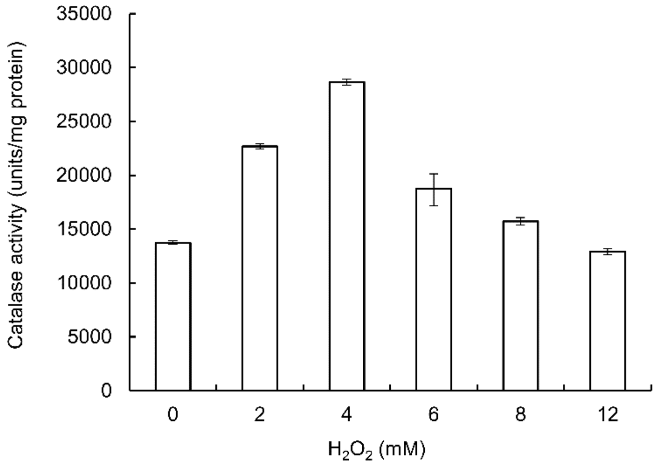

Fig. S1 Total catalase activity of A1501 treated with different H_2O_2 concentrations.

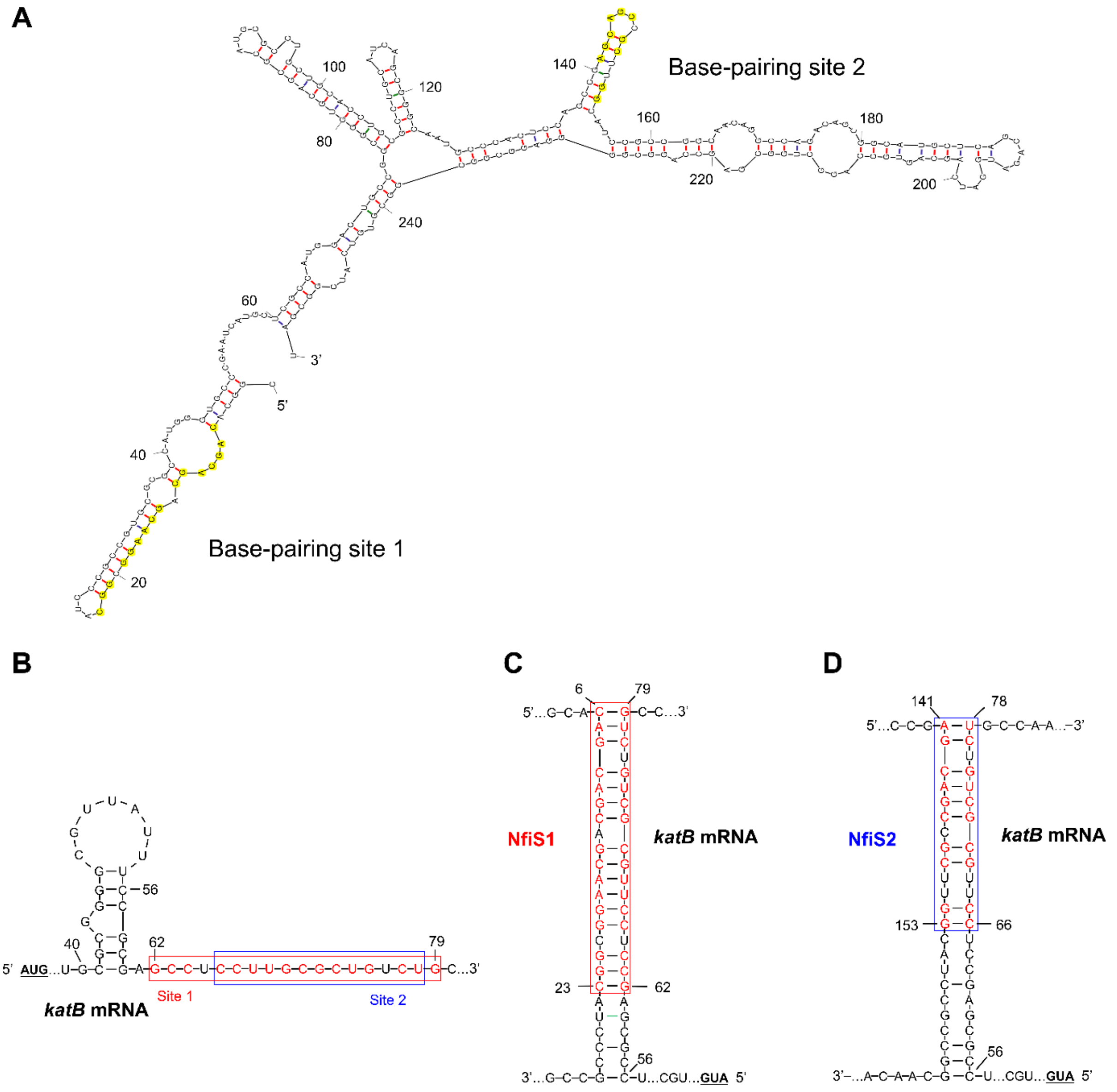

Fig. S2 Prediction of the interaction between NfiS and *katB* mRNA. (A) The predicted secondary structures of NfiS with the lowest free energy were obtained using the mfold web server. Two possible binding sites to *katB* mRNA are marked in yellow. (B) The predicted secondary structures showing the formation of a hairpin structure in the coding region of the *katB* mRNA. The *katB* mRNA residues involved in NfiS binding are shown in red. The two sequences located in the region (79 to 62 nt) of *katB* mRNA showing complementarity to predicted base-pairing site 1 (6 to 23 nt) and site 2 (141 to 153 nt) of NfiS are shown in red or

blue boxes, respectively. The two predicted sites 1 and 2 on NfiS are separated by 118 nucleotides, but predicted site 1 on *katB* mRNA completely overlaps site 2. Bases are numbered backward from the start codon. (C, D) Schematic representation of the base-pairing complex formation between *katB* mRNA and site 1 or 2 of NfiS.

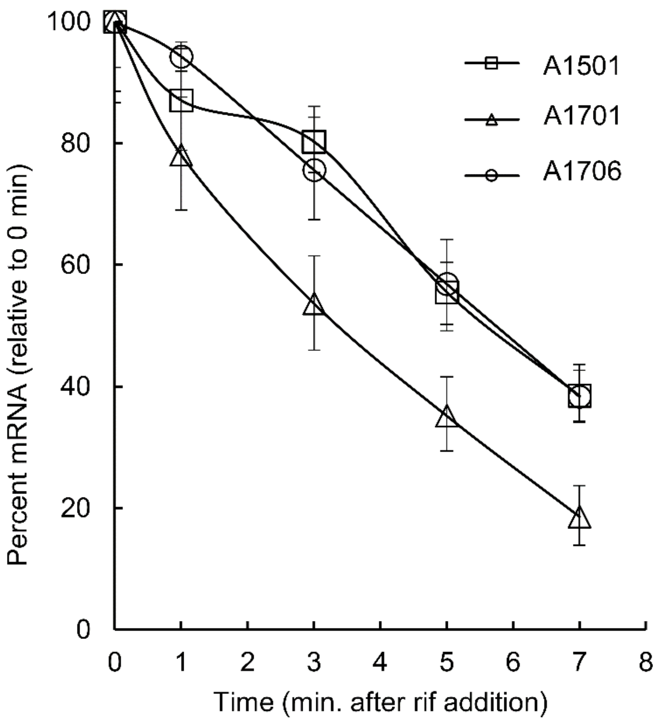

Fig. S3 Determination of the *katB* mRNA half-life in the WT A1501, Δ*nfiS* A1701, and complemented strain A1706 (complementation plasmids, namely, pLA*nfiS*-A1501) under oxidative stress conditions. The *katB* mRNA of WT was degraded with a half-life of approximately 6 min, whereas that of Δ*nfiS* was less stable and decayed with a half-life of approximately 3 min.

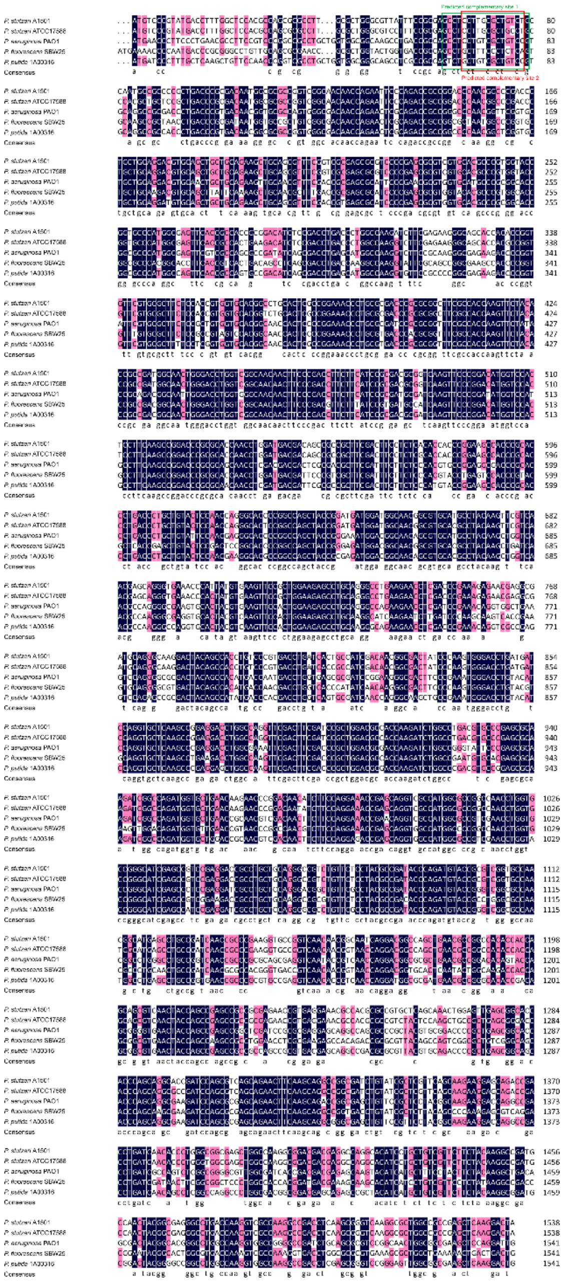

Fig. S4 Sequence alignment of the *P. stutzeri* A1501 *katB* gene with homologous sequences in *P. stutzeri* ATCC17588 (98% sequence identity with the A1501 *katB* gene), *P. aeruginosa* PAO1 (82%), *P. fluorescens* SBW25 (79%), and *P. putida* 1A00316 (83%). Predicted NfiS complementary sites 1 and 2 of *katB* mRNA are shown in green and red boxes, respectively. Nucleotides conserved at 100 and at 80% are highlighted in blue and pink, respectively. The consensus sequence is shown under the alignment.

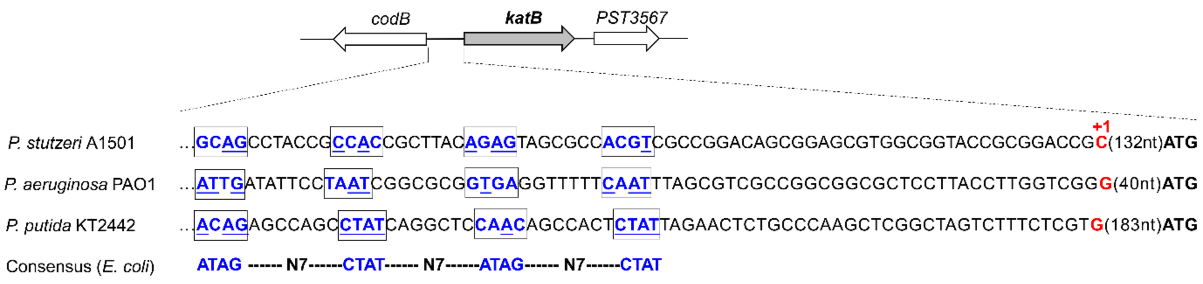

Fig. S5 Sequence alignment and comparative analysis of *katB* promoter regions in *P stutzeri* A1501, *P. aeruginosa* PAO1 and *P. putida* KT2442. Boxed residues indicate the OxyR binding sites, and the residue in red indicates the transcription starts, which were experimentally determined in *P. aeruginosa* (6) and *P. putida* (7). The consensus sequence of the *E. coli* OxyR binding site (8) is shown, and the residues matching this consensus sequence are underlined.

REFERENCES

1. Qiu YS, Zhou SP, Mo XZ, You CZ, Wang DS. 1981. Investigation of dinitrogen fixation bacteria isolated from rice rhizosphere. Chinese Sc Bull (Kexue Tongbao) 26:383-384.

2. Zhan Y, Yan Y, Deng Z, Chen M, Lu W, Lu C, Shang L, Yang Z, Zhang W, Wang W, Li Y, Ke Q, Lu J, Xu Y, Zhang L, Xie Z, Cheng Q, Elmerich C, Lin M. 2016. The novel regulatory ncRNA, NfiS, optimizes nitrogen fixation via base pairing with the nitrogenase gene *nifK* mRNA in *Pseudomonas stutzeri* A1501. Proc Natl Acad Sci U S A 113(30):E4348-E4356.

3. Staskawicz B, Dahlbeck D, Keen N, Napoli C. 1987. Molecular characterization of cloned avirulence genes from race 0 and race 1 of *Pseudomonas syringae* pv. *glycinea*.

J Bacteriol 169(12):5789–5794.

4. Figurski DH, Helinski DR. 1979. Replication of an origin-containing derivative of plasmid RK2 dependent on a plasmid function provided in trans. Proc Natl Acad Sci U S A 76:1648-1652.

5. Schäfer A, Tauch A, Jäger W, Kalinowski J, Thierbach G, Pühler A. 1994. Small mobilizable multi-purpose cloning vectors derived from the *Escherichia coli* plasmids pK18 and pK19: Selection of defined deletions in the chromosome of *Corynebacterium glutamicum*. Gene 145(1):69–73.

6. Ochsner UA, Vasil ML, Alsabbagh E, Parvatiyar K, Hassett DJ. 2000. Role of the *Pseudomonas aeruginosa oxyR-recG* operon in oxidative stress defense and DNA repair: OxyR-dependent regulation of *katB-ankB*, *ahpB*, and *ahpC-ahpF*. J Bacteriol (16):4533-4544.

7. Hishinuma S, Yuki M, Fujimura M, Fukumori F. 2006. OxyR regulated the expression of two major catalases, KatA and KatB, along with peroxiredoxin, AhpC in *Pseudomonas putida*. Environ Microbiol 8(12):2115-2124.

8. Toledano MB, Kullik I, Trinh F, Baird PT, Schneider TD, Storz G. 1994. Redox-dependent shift of OxyR-DNA contacts along an extended DNA-binding site: a mechanism for differential promoter selection. Cell 78(5):897–909.
